# Analysis of innate defences against *Plasmodium falciparum *in immunodeficient mice

**DOI:** 10.1186/1475-2875-9-197

**Published:** 2010-07-09

**Authors:** Ludovic Arnold, Rajeev Kumar Tyagi, Pedro Mejia, Nico Van Rooijen, Jean-Louis Pérignon, Pierre Druilhe

**Affiliations:** 1 Laboratoire de Parasitologie Bio-Médicale, Institut Pasteur, 28, rue du Dr Roux, 75015 Paris, France; 2 Current Address; James Mitchell Laboratory, Department of Genetics and Complex Diseases, Harvard School of Public Health, Boston, MA, USA; 3 Department of Molecular Cell Biology, VU University Medical Center, 1007 MB Amsterdam, the Netherlands

## Abstract

**Background:**

Mice with genetic deficiencies in adaptive immunity are used for the grafting of human cells or pathogens, to study human diseases, however, the innate immune responses to xenografts in these mice has received little attention. Using the NOD/SCID *Plasmodium falciparum *mouse model an analysis of innate defences responsible for the substantial control of *P. falciparum *which remains in such mice, was performed.

**Methods:**

NOD/SCID mice undergoing an immunomodulatory protocol that includes, clodronate-loaded liposomes to deplete macrophages and an anti-polymorphonuclear leukocytes antibody, were grafted with human red blood cells and *P. falciparum*. The systematic and kinetic analysis of the remaining innate immune responses included the number and phenotype of peripheral blood leukocytes as well as inflammatory cytokines/chemokines released in periphery. The innate responses towards the murine parasite *Plasmodium yoelii *were used as a control.

**Results:**

Results show that 1) *P. falciparum *induces a strong inflammation characterized by an increase in circulating leukocytes and the release of inflammatory cytokines; 2) in contrast, the rodent parasite *P. yoelii*, induces a far more moderate inflammation; 3) human red blood cells and the anti-inflammatory agents employed induce low-grade inflammation; and 4) macrophages seem to bear the most critical function in controlling *P. falciparum *survival in those mice, whereas polymorphonuclear and NK cells have only a minor role.

**Conclusions:**

Despite the use of an immunomodulatory treatment, immunodeficient NOD/SCID mice are still able to mount substantial innate responses that seem to be correlated with parasite clearance. Those results bring new insights on the ability of innate immunity from immunodeficient mice to control xenografts of cells of human origin and human pathogens.

## Background

Defences against foreign cells, including pathogens, rely on both innate or non-adaptive responses, and adaptive or antigen-specific immune responses. However, modern immunology has focused primarily or almost exclusively on the latter.

Therefore, various strains of mice having a genetic deficiency in cells responsible for adaptive immunity (*i.e*. T and B lymphocytes) have been selected, which have been used for the grafting of xenogenic cells, particularly those of a human origin. Indeed, the vast majority of these studies have focused on the grafting of human lymphocytes, haematopoietic stem cells and to a lesser extent tumor cells [1]. However, it was previously shown that one can take advantage of these immunocompromized mice to develop a mouse model for human malaria.

The initial report that bovine red blood cells (RBC) injected intra-peritoneally in SCID mice could cross the peritoneum and colonize the peripheral blood [2] was an incentive to repeat the experiment using human red blood cells (HuRBC). Although there was regrettably no understanding of the underlying mechanism of transport through the peritoneum, SCID mice harbouring up to 70 - 80% HuRBC among total RBC in mice peripheral blood were obtained [3].

However, when *Plasmodium falciparum *was injected into SCID mice, the parasites became pycnotic within hours in erythrocytes. After excluding a potential toxicity of mouse serum by *in vitro *methods, [3], it was hypothesized that innate immune defences could constitute the main limiting factor in parasite survival, and this was confirmed by employing agents able to control macrophages (MP) and other cells involved in innate defences [4,5]. Further steps were performed in an empirical manner. The model was improved by moving from the original SCID mouse to the NIHIII (Beige Xid Nude) mouse, then to the NOD/SCID mouse that have more defective innate immunity, and by identifying two components which, when combined, allowed to obtain a stable *P. falciparum *parasitaemia in some of the NOD/SCID mice [6] (namely: liposomes encapsulating dichloromethylene-diphosphonate, named clo-lip [5] and a monoclonal antibody (NIMP-14) [4] directed to mouse polymorphonuclear cells (PMN)).

Though this empirical approach demonstrated the feasibility of the objective, and could be applied to vaccine development [6,7] and drug screening [8], the control of innate defences was far from optimal in this *P. falciparum *blood stage rodent model. Indeed, the *P. falciparum *parasitaemia remained stable for extended periods of time, up to four months only in a minor subset of mice, whereas it was cleared within a few days in the majority of the animals.

The above data stress the importance of innate defences and are in agreement with long standing observations made in humans. However, despite their crucial importance innate defences have been far less studied than adaptive responses, and remain poorly known. Innate responses in *P. falciparum *infected patients were recently analysed in detail and this unveiled several dramatic modifications in the phenotypes and functions of blood monocyte/macrophage populations [9].

To complement the above descriptive analysis in humans by an experimental approach in a model, it was decided to perform in the *P. falciparum *NOD/SCID model a systematic and stepwise analysis of innate cell responses and inflammation mediators produced in response to the grafting of HuRBC, of *P. falciparum*, as well as to agents employed to control innate defences. Results bring new insights about the role and potency of innate defences against human xenografts, such as HuRBC, and human pathogens, such as *P. falciparum*.

## Methods

### Mice

Two to six months old male and female NOD/SCID mice were used. They were purchased from Charles River, and kept in an A3 animal house, *i.e*. in sterile isolators. They were housed in sterilized cages equipped with filter tops during the experimentation. Mice were provided with autoclaved tap water and a γ-irradiated pelleted diet *ad libitum*. They were manipulated under pathogen free conditions using laminar flux hoods. All animals were treated according to the French legislation.

### Human red blood cells

Human whole blood was provided by the French blood bank (Etablissement français du sang, Paris, France). Blood donors had no history of malaria and all the blood groups were used without observing any difference on parasite survival. Whole blood was washed three times by centrifugation at 900 ×g, 5 minutes at room temperature and buffy coat was separated in order to eliminate white blood cells and platelets. Packed HuRBC were suspended in SAGM (Adenine, Glucose and mannitol solution) and kept at 4°C for a maximum of 2 weeks. Before use HuRBC were washed three times in RPMI-1640 medium (Gibco/BRL, Grand Island, N.Y.) supplemented with 1 mg hypoxanthine per liter (Sigma, St Louis, MO) and warmed 10 minutes at 37°C.

### Parasite cultures

The *P. falciparum *3D7 clone was employed in this study. This parasite strain was maintained *in vitro *at 5% haematocrit in complete culture medium at 37°C in a candle jar. This medium contained RPMI-1640 medium (Gibco/BRL), 35 mM HEPES (Sigma), 24 mM NaHCO_3_, 0.5% albumax (Gibco/BRL) and 1 mg of hypoxanthine (Sigma) per liter. Cryopreserved parasites were thawed using the glycerol/sorbitol method [10] and used for the further experiments.

A non-lethal rodent parasite strain *Plasmodium yoelii *XNL1.1 was preserved in 500 μl aliquot of cryo-preserving buffer at -80°C at 22% parasitaemia. The strain was thawed at room temperature, diluted twice in RPMI-1640 medium followed by the injection of 50 × 10^6 ^parasite directly into the mice.

### Immunomodulatory agents and suppression of innate immunity

Numerous attempts were made to increase the success rate of the grafting of infected RBC. Clo-lip (provided by N. Van Rooijen) was injected through intraperitoneal (i.p.) route in order to reduce the number of tissue MP, as described previously [5]. The anti-PMN monoclonal antibody NIMP-R14 [4] was purified from a hybridoma kindly provided by Dr. M. Strath (National Institute for Medical Research, London, UK). Its activity was compared to that of two other anti-PMN monoclonal antibodies: RB6-8C5 (purified from the hybridoma kindly provided by Geneviève Milon (Institut Pasteur, Paris, France) and 1A8 (purchased from BioXcell, Lebanon). The NIMP-R14 monoclonal antibody was used in all the studies, unless specified. Various agents (all purchased at Sigma, unless specified) were used to further reduce innate immunity such as dexamethasone (1-5 mg/Kg/day), TGF-β100 ng - 1 μg/day) (PeproTech, Rocky Hill, NJ), cyclophosphamide (75 mg/kg/day), cisplatinium (1-10 mg/Kg/day), and TMβ-1 monoclonal antibody that targets NK cells (1 mg/kg/day). The effect of splenectomy and of irradiation (100 - 300 cGy) was also tested. Other experiments evaluated the addition of metabolic agents such as pABA (400 mg/kg/day), and folinic acid (400 mg/kg/day), or of antioxidants, such as vitamin E (20 mg/Kg/day; Nepalm, Cenexi, Fontenay sous bois), N-acetyl cysteine (100 mg/kg/day), trolox (4 - 100 mg/kg/day), 8-aminoguanidine (100 mg/Kg/day).

### Chemical immunomodulation protocol and mouse infection

In the current study, a previously described immunomodulation protocol was employed [11], modified as described in [12]. On day -13, each mouse received a dose of 10 mg/kg of mAb NIMP-R14 by i.p. injection. On day -12, each mouse received 0.2 ml of the suspension of clo-lip by the same route. On days -9, -6, -3 each mouse received 0.5 ml of HuRBC i.p. mixed with a dose of 10 mg/kg of mAb NIMP-R14 and 0.2 ml of clo-lip. On day 0 mice were infected with 500 μl HuRBC parasitized by *P. falciparum *at a parasitaemia of 1% (all the developmental forms, *i.e*. trophozoite, schizont and rings, were present) mixed with a dose of 10 mg/kg of NIMP-R14 antibody. Afterwards, a dose of 10 mg/kg of antibody NIMP-R14, 0.2 ml of clo-lip and 0.5 ml of HuRBC was injected i.p. at 3 days interval, until the end of the study. The follow-up of the infection was performed by daily Giemsa stained thin blood films drawn from the tail vein.

### Haematological parameters and grafting of *P. falciparum*-HuRBC

The study blood samples were collected from mice retro-orbital sinus on heparin. Various haematological parameters such as haematocrit, leukocyte number and phenotype (Ly-6C APC, Ly-6G APC (Miltenyi Biotec, Germany), CD115 PE, CD43 FITC, CD62L FITC, CD11b FITC, DX5 FITC, CD122 PE (BD Biosciences, UK) in peripheral blood samples were monitored, as well as the phenotype characterization of monocytes (CD11b^+^, CD115^+^), inflammatory monocytes (CD43^-^, CD62L^+^, Ly-6C^+^), PMN (CD11b^+^, ly6G^+^), and natural killer cells (DX5^+^, CD122^+^). Total leukocyte number (leukocytes/μl blood) was evaluated by lysing 20 μl of total blood with BD FACS™ Lysing solution, and counting on Malassez haematocytometer. Since successfully grafted mice have a significant, but variable, percentage of HuRBC in their peripheral blood, parasitaemia in mice was expressed as the overall percentage of *P. falciparum *infected RBC among total RBC, *i.e*. both human and mouse RBC observed in thin blood smears. In addition, the peritoneal blood parasitaemia was measured in the smears drawn from the peritoneum. The percentage of HuRBC in the peripheral blood of mice was determined by an immunofluorescence technique using a FITC labeled anti-human glycophorin A monoclonal antibody (DAKO, Denmark).

### Assay of cytokines and chemokine

100-150 μl blood samples were collected from the retro-orbital sinus with the help of Pasteur pipette, and sera were stored at -80°C. Cytokines (IL-6, MCP-1, IFNγ, TNFα, IL-12p70 and IL-10) were quantified using the BD™ Cytometric Bead Array mouse inflammatory kit following the manufacturer's recommendations.

### Statistical analysis

The paired test was used for statistical analysis. P values of less than 0.05 were considered significant. In the results section only differences reaching significance are mentioned.

## Results

### Various patterns of peripheral blood parasitaemia are observed in NOD/SCID mice

**Figure 1:**
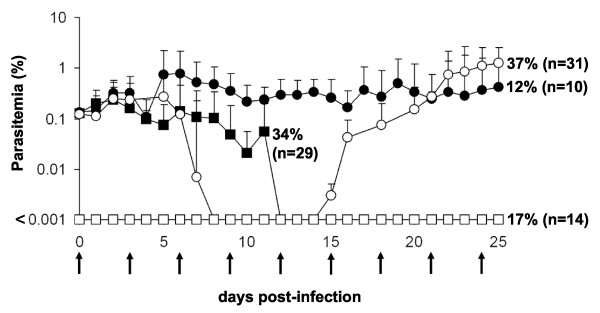
**Patterns of blood parasitaemia observed in NOD/SCID mice**. In four independent experiments, a total of 84 NOD/SCID mice were infected with a single i.p. infection of *P. falciparum *3D7 strain on day 0 and HuRBC (500 μl of pellet at 50% haematocrit), clo-lip (200 μl) and NIMP-R14 (10 mg/kg) antibody were injected i.p. every 3 days as previously described (black arrows). The preparatory phase prior to the injection of infected HuRBC is not shown. Parasitaemia was measured on tail blood smears by counting the number of infected RBC among 10000 total human and mouse RBC. Parasitemia lower than 0,001 are not considered. The figure shows the average parasitaemia ± SD, and the percentages (and number of mice) corresponding to each pattern of parasitemia.

Although stable long-lasting parasitaemia could be obtained and employed for various applications [8,11] the pattern of parasitaemia has never been homogeneous, i.e. varied greatly from one animal to the other, and the various attempts to modify the protocol failed to improve results significantly. For instance, among a total of 84 mice studied recently under rigorous and well-controlled conditions using the standard immunomodulation protocol described above, four different patterns of peripheral blood parasitaemia could be described (summarized in Figure 1). Following a single infection by *P. falciparum*, 17% of mice remained parasitologically negative, 34% showed a transient parasitaemia lasting for *ca*. 12 days post-infection, 12% showed a stable parasitaemia for more than 20 days and 37% showed an almost total parasite clearance from peripheral blood, however followed by a re-emergence a few days later, without new parasite inoculation, *i.e*. a second wave of parasitaemia lasting for the life-span of the animal. The sequence of events of the latter pattern considered the most informative, was selected for further analysis.

Various modifications to the standard protocol were assessed, and resulted only in differences in the proportion of mice presenting the four patterns of parasitaemia described above. These modifications include: 1) variations in the dose and schedule of administration of the different components (*i.e*. infected and uninfected HuRBC, clo-lip and NIMP-R14, including modifications proposed by others [12]); 2) use of various agents to reduce innate immunity, such as cyclophosphamide, cisplatinium, irradiation, dexamethasone, TGF-β, splenectomy, TMβ-1 antibody targeting NK cells 3) Addition of metabolic precursors such as pABA, folinic acid to supply nutritive factors for the parasite 4) Addition of antioxidants such as vitamin E, N-acetyl cysteine (NAC), trolox, 8-aminoguanidine. These empirical approaches never brought a significant or reproducible improvement of parasite survival in NOD/SCID mice.

These results raise the question of which factor(s) are critical to control in order to obtain a stable parasitaemia and prompted the launch of a detailed analysis of the remaining innate immune defences, mainly MP and PMN.

### The strong inflammation induced by *P. falciparum *is associated with parasite clearance

In mice showing a recrudescence (fourth pattern described above, which was thought to be the most discriminating, see Figure 1) the following parameters were analysed in kinetic manner: 1) the numbers and phenotype of blood leukocyte subsets, 2) various cytokines serum levels, 3) HuRBC grafting in the peripheral blood, together with 4) parasitaemia.

**Figure 2:**
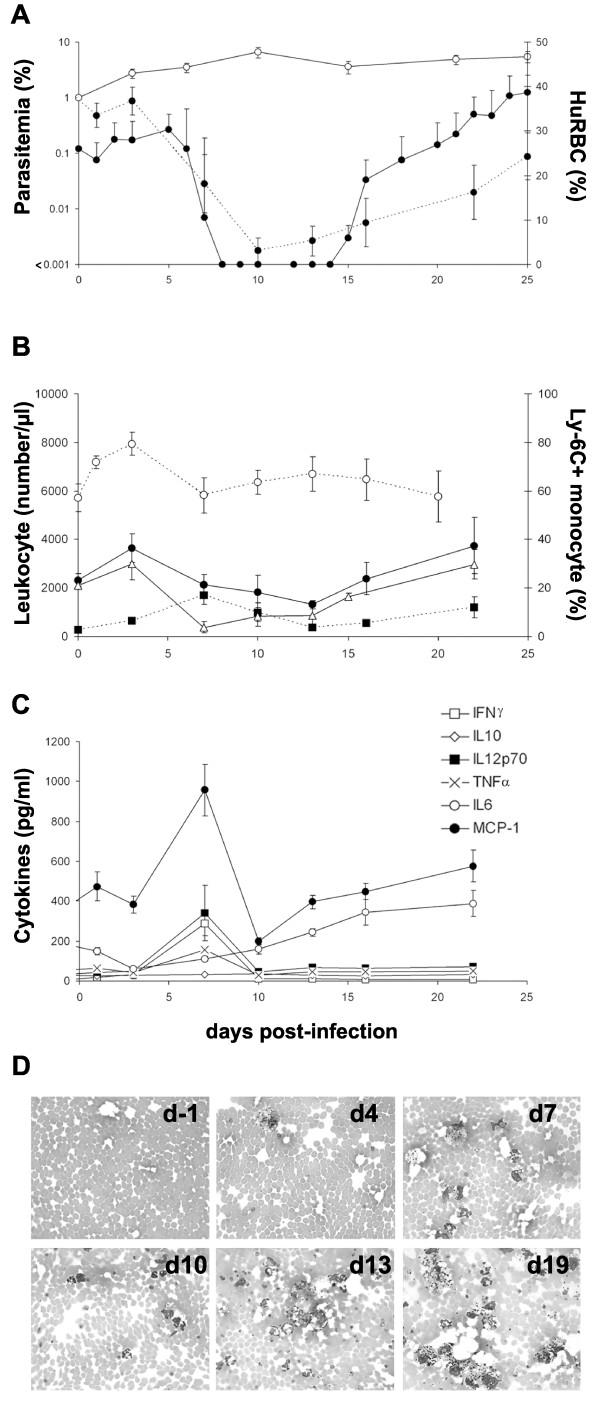
***P. falciparum *is highly pro-inflammatory**. **(A) **Peripheral (plain circles) and peritoneal (open circles) parasitaemia (average of 31 mice ± SEM) counted on blood smears and percentage of human glycophorin A+ RBC grafted in mouse peripheral blood detected by FACS among total RBC (full circle, dotted line). Parasitemia lower than 0,001 are not considered. **(B) **Total number of leukocyte per μl of blood (plain circle) at different times post-infection. The numbers of CD11b^+ ^F4/80^+ ^monocytes (black square, dotted line) and CD11b^+ ^Ly-6G^+ ^PMN (white triangle) were calculated from the percentages obtained by FACS analysis. The percentage of CD43^- ^CD62L^+ ^Ly-6C^+ ^inflammatory monocytes was also assessed by FACS (open circle, dotted line). **(C) **Cytokines/chemokine levels were estimated using the CBA mouse inflammatory cytokines kit by FACS. **(D) **Representative peritoneal blood smears showing the progressive phagocyte recruitment following *P. falciparum *infection. Results represent the means ± SEM from 3 distinct experiments (n = 14 mice).

Results, summarized in Figure 2A, show that, whereas parasitaemia in peritoneal blood remains stable over time, conversely, peripheral blood parasitaemia decreases markedly. This decrease is related to a major clearance of HuRBC in this compartment, despite repeated blood injections, which is parasite-dependant since the numbers of circulating HuRBC in control, uninfected mice, remain stable (see below).

The parasite was found to induce a very potent pro-inflammatory effect characterized by a transient increase in peripheral leukocytes numbers mostly monocytes (MO) (1690 ± 1080 MO/μl on day 7 post-infection *vs *290 ± 125 on day 0, *i.e*. 5.8 fold increase; P < 0.006) and to a lesser extent PMN (3000 ± 1500 PMN/μl on day 3 post-infection *vs *2100 ± 750 on day 0, *i.e*. 1.4 fold increase; not significant) (Figure 2B). Moreover, during the first few days following the infection, a transient increase (Figure 2B) in blood MO having an inflammatory phenotype CD43^- ^CD62L^+ ^Ly-6C^+ ^was observed (57.2 ± 12.7% of MO on day -1 before infection *vs *80 ± 11% on day 3 post-infection; P < 0.03) (the Gr-1 antigen is not expressed on NOD/SCID MO surface). In addition, the parasite induces a major recruitment of leukocytes in the peritoneal cavity as compared to controls receiving uninfected HuRBC (Figure 2D).

The peak of blood MO was associated with a peak of secretion of several inflammatory cytokines such as IL-12p70 (342 ± 520 pg/ml on day 7 post-infection *vs *33 ± 6.5 pg/ml on day -1 before infection), IFNγ (290 ± 215 pg/ml on day 7 *vs *5 ± 2.2 pg/ml on day -1) and TNFα (155 ± 53 pg/ml on day 7 *vs *53 ± 26 pg/ml on day -1) or chemokine such as MCP-1 (from 355 ± 290 pg/ml on day -1 to 960 ± 480 pg/ml on day 7 post-infection) (P < 0.009).

Results attest of the strong inflammation induced by the parasite; however the latter is transient, (Figure 2C) and accordingly the number of leukocyte decreases to reach values close to the steady state.

The decrease in parasite-induced inflammation was followed by a new increase in HuRBC numbers in peripheral blood, which understandably correlated with a recrudescence of *P. falciparum *blood parasitaemia. This second wave of parasitaemia itself induced an increase of leukocyte counts, of IL-6 and MCP-1, however not of IL-12p70, IFNγ and TNFα, and in addition, was not associated with a re-emergence of inflammatory MO. Hence, the secondary parasitaemia was less pro-inflammatory than the first. This also point to an important role for inflammatory MO in the initial clearance of uninfected and *P. falciparum *infected HuRBC.

### *Plasmodium yoelii *is far less pro-inflammatory than *P. falciparum*

**Figure 3:**
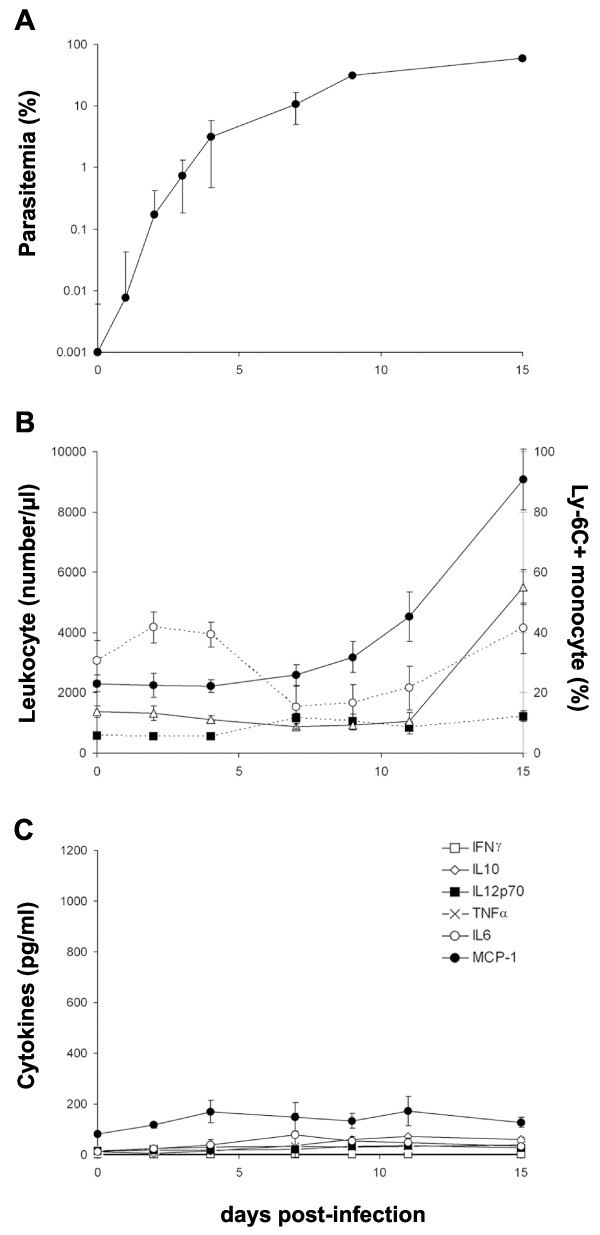
**P. yoelii is far less pro-inflammatory than P. falciparum**. *P. yoelii *non lethal XNL 1.1 strain was injected i.p. in NOD/SCID mice and the same parameters as in *P. falciparum *infected mice were assessed (see the legend of figure 2). **(A) **Peripheral blood parasitaemia; **(B) **Total number of leukocyte per μl of blood (plain circle) at different time post-infection. The numbers of CD11b^+ ^F4/80^+ ^monocytes (black square, line dot) and CD11b^+ ^Ly-6G^+ ^PMN (white triangle) were calculated from the percentages obtained by FACS analysis. The percentage of CD43^- ^CD62L^+ ^Ly-6C^+ ^inflammatory monocytes was also assessed by FACS (open circle, dotted line); **(C) **Cytokines/chemokine levels determined using the CBA mouse inflammatory cytokines kit. Results represent the means ± SEM from 2 distinct experiments (n = 5 mice).

Innate immune responses triggered by *P. falciparum *were compared with those elicited by the murine non-lethal parasite *P. yoelii *XNL 1.1 strain. In contrast to *P. falciparum*, *P. yoelii *induced an exponential increase in parasitaemia in immunodeficient NOD/SCID mice reaching more than 60% within two weeks (Figure 3A), accompanied by a major drop in haematocrit (52 to 18% by week 2), and ultimately led to the death of the animals. An increase in the numbers of circulating leukocytes occurred, but was delayed, until parasitaemia was already high (*ca*. 10%) (Figures 3A and 3B). The number of leukocytes increased 3.5 fold from day 7 (2600 ± 800 leukocytes/μl) to day 15 (9100 ± 2250) post-infection (P < 0.004). This was mainly due to a 4.2 fold increase in PMN from day 11 (1060 ± 630) to 15 (5500 ± 1300) (P < 0.008). There was a major, 7.1 fold, increase of NK cells from day 7 (95 ± 50 NK cells/μl) to day 15 (675 ± 180) (P < 0.001), and a lower, 2.2 fold, increase in MO from day 4 (560 ± 230 MO/μl) to 7 (1200 ± 390) (P < 0.013).

*Plasmodium yoelii *infection did not induce the strong inflammation characteristic of *P. falciparum*. Only MCP-1 was detected to a significant level (145 pg/ml till the end of the study) but its basal level was already elevated (80 pg/ml) (Figure 3C). The other cytokines assayed did not show any significant increase as compared to basal level (Figure 3C). Moreover, *P. yoelii *did not induce the strong increase of CD43^- ^CD62L^+ ^Ly-6C^+ ^inflammatory MO which was very significant with *P. falciparum *(Figure 2B), but only several moderate variations (i.e. a small initial increase from 30.6 ± 6.5% on day 0 to 41.7 ± 5.2% on day 2 post-infection (P < 0.0045) followed by a temporary decrease, 15.2 ± 7.3% on day 7 post-infection (P < 0.0045) then by a new increase to reach steady state value on day 15 post-infection) (Figure 3B). In the absence of adaptive immune responses, all mice infected with *P. yoelii *died between day 16 to day 20 post-inoculation (not shown).

### HuRBC, and anti-inflammatory agents, induce low-grade inflammation

**Figure 4:**
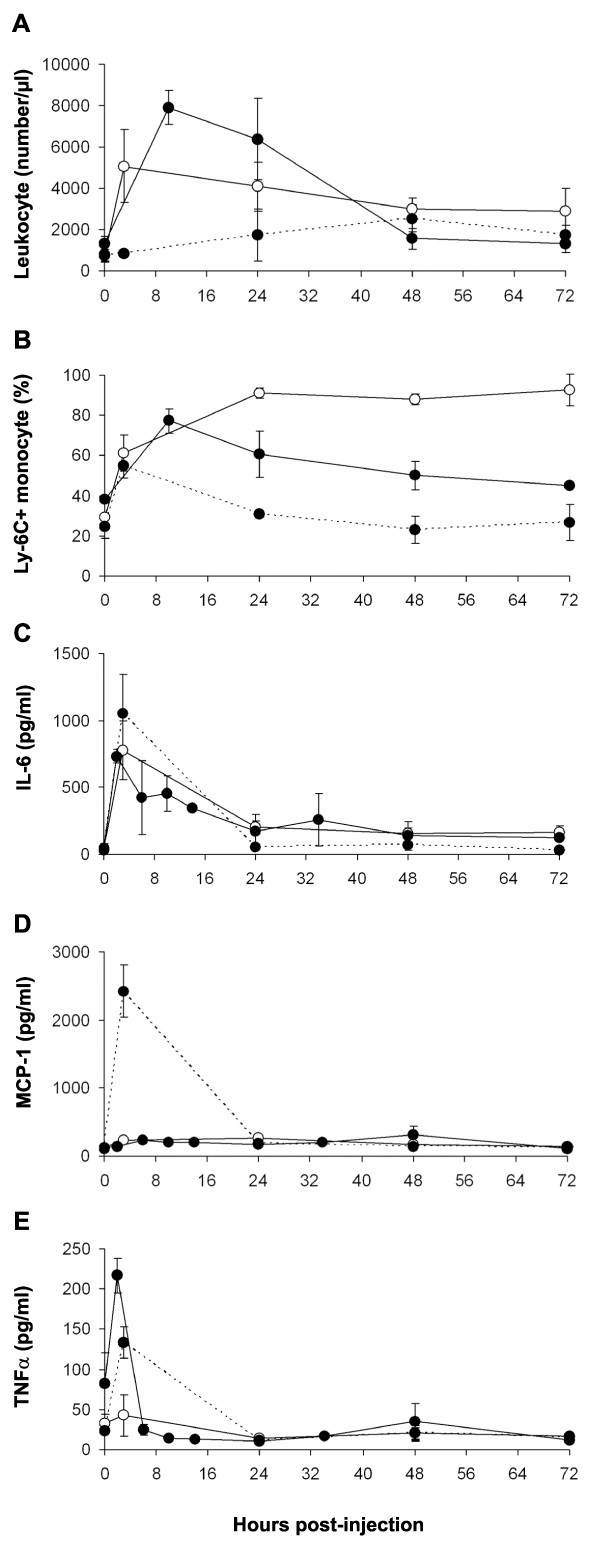
**HuRBC, clo-lip and NIMP-R14 antibody are pro-inflammatory**. HuRBC (full circle, plain line), clo-lip (open circle) and NIMP-R14 antibody (full circle, dotted line) were injected once in the peritoneum at hour 0, at doses identical to those used for the whole immunomodulation protocol. Leukocyte number **(A)**, CD43^- ^CD62L^+ ^Ly-6C^+ ^inflammatory monocytes **(B) **and inflammatory cytokines/chemokine **(C-E) **were assessed in mouse peripheral blood by FACS. Results are the means ± SD. from groups of 4 mice.

Considering the indications that inflammation was related to the clearance of the parasite, it was decided to examine whether HuRBC, clo-lip and NIMP-R14 antibody also induce inflammation. The effects of each component, injected i.p. upon 1) the number and phenotype of blood leukocytes, and 2) cytokines serum levels, were examined. The inflammatory effect of HuRBC was not unexpected, as it represents a heterologous graft. The i.p. injection of HuRBC induced an increase in leukocyte numbers (Figure 4A) (P < 0.003 between day 0 and day 3) mainly PMN and to a lesser extent MO and NK cells (data not shown). The inflammatory MO subset increased transiently to peak at 76% of total MO 10 hours post infection (P < 0.009), and then progressively decreased to basal level (Figure 4B). HuRBC grafting also led to a transient secretion of IL-6 (732 ± 51 *vs *47 ± 14.7 pg/ml; P < 0.003) and TNFα (216 ± 51 pg/ml *vs *75 ± 79 pg/ml; P < 0.1) (Figures 4C and 4E), without substantial modifications of MCP-1, IL-10, IL-12p70 or IFNγ.

Unexpectedly, it was observed that clo-lip induce a fast and strong inflammatory reaction. Indeed, as soon as three hours post liposomes injection, the number of circulating leukocyte increased by 6.8 fold (Figure 4A) (MO 3.14 fold, PMN 8.8 fold and NK 3.07 fold) (P < 0.01), and thereafter, decreased but slowly (remaining four times higher than basal level 48 hours post-injection). In addition, clo-lip induced an increase of the "inflammatory" MO subset (CD43^- ^CD62L^+ ^Ly-6C^+^) that lasted for more than three days (Figure 4B) (P < 0.001). Clo-lip injection also led to a transient release of IL-6 (777 ± 445 *vs *48 ± 30 pg/ml at 3 hours post-injection) (Figure 4C) (P < 0.03).

Finally, the anti-PMN monoclonal antibody NIMP-R14 had a modest effect on the number of leukocytes and on the percentage of inflammatory MO, but was the strongest inducer of IL-6 (1054 ± 292 *vs *30 ± 7 pg/ml at 3 hours post-injection) and of MCP-1 (2425 ± 760 pg/ml at 3 hours post-injection) (Figure 4C and 4D), and also induced the release of TNF(133 ± 19 *vs *24 ± 3.7 pg/ml at 3 hours post-injection) (Figure 4E) (P < 0.04).

Altogether these results indicate that the immunomodulators clo-lip and NIMP-R14 antibody, which have previously been shown to be indispensable to the grafting of *P. falciparum*, actually reduce innate responses, though at the expense of a transient but substantial inflammation.

### The repeated injection of HuRBC, clo-lip and anti-PMN reduces inflammation and improves HuRBC grafting

**Figure 5:**
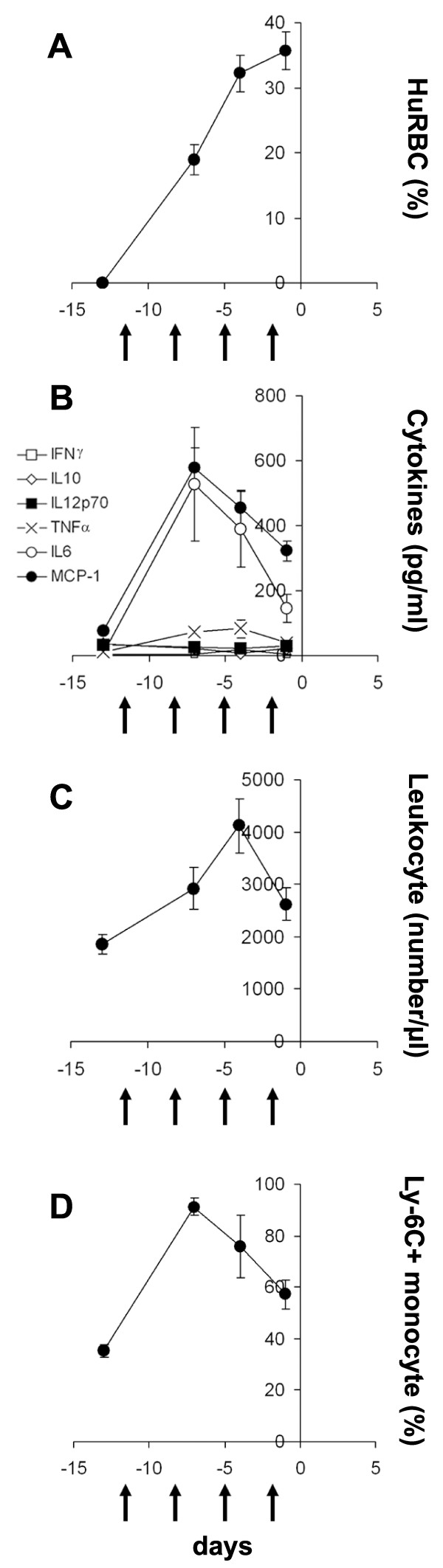
**Graft of HuRBC and markers of inflammation during the immunomodulation protocol**. **(A) **The percentage of HuRBC grafted in mouse peripheral blood was assessed by FACS by using an anti-human glycophorin A antibody, after several co-injections of HuRBC, clo-lip and NIMP-R14 antibody into the peritoneum (black arrows). **(B) **Cytokines/chemokines were assessed in mouse sera by FACS using the CBA mouse inflammatory cytokines kit. **(C) **Leukocyte number. **(D) **CD43^- ^CD62L^+ ^Ly-6C^+ ^inflammatory monocytes were assessed by FACS. Results are means ± SEM of 2 experiments (n = 15 mice).

The intriguing results above led to analyse the events spanning the 13 days of the immunomodulation protocol preceding the injection of the parasite. This protocol consists in four co-injections of HuRBC, together with clo-lip and NIMP-R14 antibody, to prepare the host to the infection as suggested by others [12]. A progressive decrease of inflammation during the immunomodulation protocol was observed. At the end, the levels of cytokines were lower (Figure 5B) than those induced by a single injection, for IL-6 (146 ± 43 *vs *527 ± 175 pg/ml), MCP-1 (322 ± 31 *vs *580 ± 58 pg/ml), and TNF(40 ± 12 *vs *72 ± 12 pg/ml) (P < 0.03). The number of leukocytes and the percentage of CD43^- ^CD62L^+ ^Ly-6C^+ ^MO also decreased over time (Figures 5C and 5D) (P < 0.05). These changes resulted in an improved grafting of HuRBC as compared to a single injection (35.6 ± 14.3% *vs ca*. 10%) (Figure 5A).

### MO/MP are critical in controlling *P. falciparum *and HuRBC grafting in NOD/SCID mice

An attempt was made to identify the cell subset most critical in controlling *P. falciparum *in NOD/SCID mice. NK cells are less numerous and functionally deficient in NOD/SCID as compared to BXN mice. In addition, their depletion by the TMβ-1 antibody did not result in any improvement. Both observations do not point to a major role of these cells (data not shown).

The rise in leukocyte numbers induced by *P. falciparum *is predominantly due to an important increase in PMN numbers, leading to legitimately suspect their involvement. To explore the role of PMN, three different monoclonal antibodies were employed, that differ in their efficiency to deplete PMN, namely 1A8, RB6-8C5 and NIMP-R14 antibodies. 1A8 antibody led to a major depletion of PMN for a period of ten days after infection, whereas the other two had only a moderate effect. Despite these differences, the resulting parasitaemia were essentially similar, which does not designate PMN as a main effector against infected HuRBC (Additional file 1).

**Figure 6:**
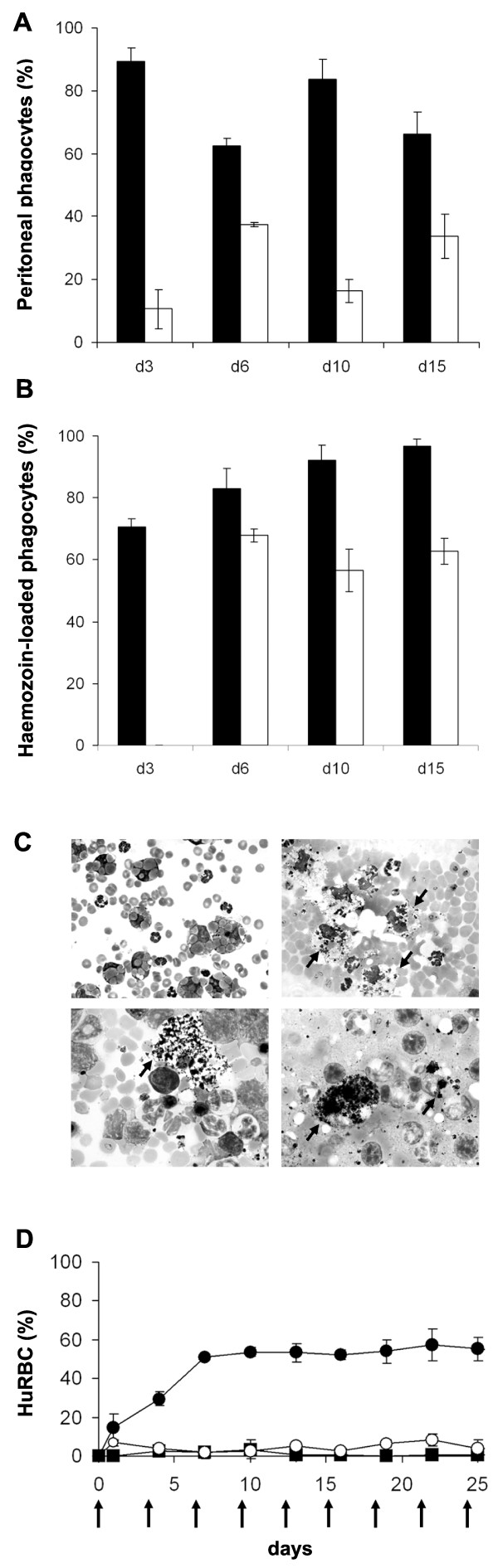
**Evaluation of the role of PMN, and of monocytes/macrophages, in the graft of infected and non-infected HuRBC**. A peritoneal smear was drawn at different times post-infection in order to assess the phenotype of peritoneal phagocytes. **(A) **MP (black bar) and PMN (white bar) were identified by microscopy in order to determine the percentage of each subset among the total number of phagocytes counted on the smear (at least 500). **(B) **Percentages of MP (black bar) and PMN (white bar) having ingested at least one particle of malaria pigment. Results are the mean ± SD from 4 infected NOD/SCID mice. **(C) **Representative Giemsa staining of different organs smears performed 45 days post-infection from 3 different NOD/SCID mice (upper panel left and right: peritoneum; lower left panel: spleen, lower right panel: liver). Arrows indicate haemozoin loaded MP. **(D) **Effect of clo-lip and NIMP-R14 antibody on HuRBC grafting in NOD/SCID peripheral blood. HuRBC are injected alone (Black square) or in combination with clo-lip (plain circle) or with NIMP-R14 antibody (open circle). Black arrows represent the days of injection. Results are means ± SEM of 2 experiments (n = 6 mice per group).

The above results led to address the role of MO and of the large MP derived from them. Firstly, MP is the main subset of phagocytes recruited into the peritoneum all over the course of infection (Figure 6A) (P < 0.05). Secondly, they were found to be the most active, and in particular more active than PMN at ingesting both infected and non-infected HuRBC in the peritoneum (Figure 6B and 6C upper panel) but also in the spleen (Figure 6C lower left panel) and in the liver (Fig 6C lower right panel). Moreover, clo-lip target only circulating MO and MP, and Figure 6D clearly shows that clo-lip is necessary for the grafting of HuRBC: the percentage of circulating HuRBC was 54 ± 2% after three clo-lip injections *vs *0.95 ± 0.93% without, or 4.1 ± 2% with the anti-PMN NIMP-R14 antibody. Altogether, these results point to the MO/MP lineage as the most efficient component in controlling the graft of *P. falciparum *infected HuRBC.

## Discussion

Results confirm that innate defences are potent against xenografts and pathogens in mice lacking T, B and NK cell functions, and provide an insight of their effect against *P. falciparum *in immunodeficient mice. Several years ago, our group has taken advantage of immunocompromized mice, *i.e*. mice genetically defective in their adaptive immunity, to generate a *P. falciparum *mouse model, which could be successfully used for several types of applications such as assessment of antibodies directed to new vaccine candidates [6,7,11] or of anti-malarial drugs [8] with any *P. falciparum *strain or isolate [12]. The repeated injection into the peritoneum of HuRBC, clo-lip and NIMP-R14 antibody allowed *P. falciparum *growth in mouse peripheral blood, but at the expense of a high failure rate, since only a subset harboured a stable parasitaemia (*i.e*. 12% among several hundred NOD/SCID mice tested have a stable parasitaemia and 37% have a recrudescent parasitaemia). Therefore, it was decided to focus further work on the analysis of murine innate immune defences induced by this very pro-inflammatory parasite in order to gather an understanding, *i.e*. to attempt to identify the critical defence component(s) limiting successful grafting.

The present study shows that 1) *P. falciparum *induces a major inflammation characterized by an increase in peripheral leukocytes and by the release of inflammatory cytokines in the serum; 2) parasitaemia in the peritoneum remains stable whereas the decrease observed in the peripheral blood is related to HuRBC clearance in this compartment, triggered by the parasite; 3) *P. yoelii*, induces a very moderate inflammation as compared to *P. falciparum*, suggesting that a parasite adapted to its host does not trigger the same inflammatory response (which may be related to host factors, for example a lower recognition by host receptors, and/or to parasite factors, for example a lower production of pro-inflammatory molecules presented by this parasite); 4) clo-lip, NIMP-R14 and HuRBC also induce an inflammation, but far less important than that triggered by the parasite itself, and the former is anyhow essential to parasite grafting; 5) none of the additional immunosuppressive, anti-inflammatory, anti-oxidant and nutritive factors assessed in empirical manner was able to significantly, or reproducibly, improve *P. falciparum *survival; and 6) MP seem to bear the most critical function in controlling *P. falciparum *in these mice.

The innate immune defences that remain in immunocompromized mice have seldom been analysed. Surprisingly, only one study has so far focused on this issue. It reported the occurrence of a major inflammation in relation to human leukocytes grafting in SCID mouse peritoneum [13]. It consists in a massive and early (24 h) recruitment of neutrophils and in the secretion of a wide spectrum of murine cytokines, such as IL-6, TNF, IFNγ, IL-1β, IL-10, IL-12p40 in the peritoneal cavity. The role of granulocytes is supported by improved engraftment of human leukocytes following administration of the anti-granulocyte antibody RB6-8C5 [14]. Since this report, innate immune responses in immunocompromized mice have remained surprisingly little explored, despite the general agreement that they are very efficient in defence against xenografts [15,16]. Even NOD/SCID/IL-2rγ^-/- ^mice, which present substantial additional defects in innate immunity (they lack functional T, B and NK cells, have reduced functions of MP and dendritic cells, and several cytokines pathways are totally impaired) still require the use of pharmacological agents or irradiation/splenectomy to allow for the grafting of cells of human origin [15,17].

The present study model includes a "double xenograft", of HuRBC and of *P. falciparum*. A first limitation of this model is the lack of understanding of the transperitoneal passage of both uninfected and infected HuRBC in mouse peripheral blood, and its lack of reproducibility (17% of mice were negative). The i.p. route of administration of HuRBC was chosen as it was previously found effective using bovine RBC [2], as it constitutes a reservoir whereas HuRBC injected i.v. are quickly eliminated [18]. However, the mechanism of passage being unknown, though there are indications for lymphatic drainage [19,20], the reasons for occasional blockade are even less clear. There might be a pure mechanical component as the simple increase in HuRBC volume (2 ml at 50% haematocrit daily) led us and others [21] to improve HuRBC chimerism in peripheral blood. Secondly, the increase of clearance of HuRBC (both infected and non-infected) 7 to 10 days after injection of the parasite, is likely inflammation dependent as it was found to be associated with a concomitant peak of inflammatory cytokines and chemokine (IL-12p70, IFNγ, TNFα and MCP-1) in mouse serum. TNFα is known to increase erythrophagocytosis and to induce anaemia during malaria both in mouse and in human [22]. Splenectomy improves HuRBC grafting, suggesting that splenic MP play a role in this clearance [21,23]. Thirdly, in those rare cases (5%) when the peritoneum cavity was invaded by millions of MP this resulted in HuRBC phagocytosis, and clearance of the graft in this compartment.

Together these data suggest a prominent role of MP in the rejection of both uninfected and infected HuRBC by two main mechanisms: the release of inflammatory mediators reflecting the activation of MO and the resulting increased erythrophagocytosis. The role of MP in xenograft rejection is well documented [24]. For instance they account for the majority of infiltrating leukocytes during the rejection of pig-to-primate xenografts [25,26] and selective MP depletion in immunocompetent rodents resulted in significant delays in cellular infiltration and xenograft rejection [27,28]. Finally, clo-lip which depletes MO/MP was clearly essential to both HuRBC and *P. falciparum *survival, stressing also in this model the importance of MP.

The mechanism of HuRBC phagocytosis by murine MP is yet to be elucidated. It can not be due to complement or by Fc receptors-mediated opsonization, as NOD/SCID mice have neither complement activity [29] nor antibodies [29,30]. C-type lectins integrated in MP membrane have been reported to mediate erythrophagocytosis by directly binding to sugar ligands on the surface of senescent or altered RBC [31,32]. Moreover, the signal regulatory protein a (SIRPα) is a critical immune inhibitory receptor on MP that reacts with CD47 to prevent autologous phagocytosis. Since CD47 is species specific, healthy HuRBC may be phagocytozed by murine MP due to their inability to induce murine MP SIRPα tyrosine phosphorylation [33,34]. Conversely, phagocytosis of *P. falciparum *infected HuRBC by MP is well documented, and the scavenger receptor CD36, which recognizes PfEMP-1 on the surface of infected RBC, is widely implicated in this process. Indeed, *P. falciparum *phagocytosis decreases by 80% using blocking antibodies or CD36^-/- ^murine MP [35,36]. MP also possess a large array of pathogen-recognition receptors such as toll-like receptors (TLRs) that recognize *P. falciparum *GPI anchors, that are considered as key malaria pathogenicity factors [37,38]. TLR-2 and to a lesser extent TLR-4 directly recognize infected RBC [39] whereas TLR-9 recognize only the haemozoin pigment [40-42], but all trigger inflammation through the MyD88/NFκB pathway. Uric acid (derived from hypoxanthine accumulated by the parasite) has been recently reported to be another major inflammatory mediator involved in *P. falciparum *infection [43]. The effect of haemozoin pigment on MO/MP is debated as it was reported to impair their function such as blocking of phagocytosis [44-46], but also to be a potent proinflammatory agent. Indeed it can trigger the release of NO, TNF, IL-6, IL-1β, MIP-1α, MIP-1β, MIP-2, MCP-1 and of many other mediators after *in vivo *administration [47-49]. Successive effects, *i.e*. pro-inflammation followed by a relative anergy, may explain this apparent contradiction. Whatever the complexity of the inflammatory response induced by the parasite, it probably explains the present observation, also made by another group, that the injection of infected HuRBC induces a decrease of the number of circulating HuRBC, either infected or not. The results of the present study suggest that, whereas uninfected HuRBC induce a moderate inflammation, the parasite is a far more potent pro-inflammatory component leading to a significant phagocytosis of HuRBC (both infected and uninfected).

The remarkable efficiency of the innate immune system to control plasmodium infection is in keeping with observations in humans: indeed, the parasitaemia recorded in a primary malaria attack, in non-immune travellers, is usually quite modest, 0.1% on average (PLD, unpublished observations in 700 infected European travellers), whereas the theoretical 16×/48 h multiplication rate would lead to heavy parasitaemia in the absence of strong innate defences. Results in NOD/SCID show similarities and differences with *P. falciparum *activation of innate defences in humans. Recent studies performed in *P. falciparum *infected and exposed individuals have shown a strong pro-inflammatory effect with an overall increase in intermediate and inflammatory MO expressing CD16^+^, mIFNγ and 2- to 10-fold increase in serum mediators, such as IL-6, IL-10, IFNγ, TNF and MCP-1 [9]. However, two opposite phenotypes could be identified in humans, based on chemokine receptors expression, which corresponded to differences in activity in the antibody-dependent, MO-mediated ADCI mechanism of defence and with 10 fold differences in the resulting parasitaemia in humans [9]. These two opposite phenotypes can correspond either to true differences between two groups of individuals or, alternatively, to successive states of MO activation by the parasite at different time-points. One of the patterns observed in NOD/SCID mice, where parasitaemia and inflammation were followed by a decrease of both, brings support to the latter view and is reminiscent of the two states described in mouse MO exposed to haemozoin. Finally, the observation that the rodent parasite *P. yoelii *induces much less inflammation than the human parasite in mice challenges the general assumption that the evolution-driven adaptation of Plasmodium to their respective hosts depends mainly on the adaptive immune system, and indicates that the role of non-adaptive immunity should be taken in consideration. The fine molecular tuning of parasite molecules required for the adaptation through the co-evolution of the parasite with their usual host has most likely taken place for molecules interacting with the adaptive immune system ("antigens") and for molecules interacting with the innate defences. The numerous differences between *P. yoelii *and *P. falciparum *observed in our study bring support to this hypothesis.

More generally, the results of the present study stress that a deficiency in adaptive immunity is far from being enough to ensure the success of xenografts. It is also essential to control innate defences (which can not be knocked out safely). In this respect, *P. falciparum*-SCID mice constitute a convenient model as the effect of innate defences is readily visible within hours, in contrast with other types of grafts (*e.g*. lymphocytes). However, innate immunity proved as efficient as it is difficult to control. Three strategies have been used for this purpose. Firstly, splenectomy improved *P. falciparum *survival in NOD/SCID mice [18]. Secondly, the suicide-based approach developed by Van Rooijen with clo-lip [5], despite its paradoxical inflammatory effect, was nonetheless efficient at depleting MP. Clo-lip proved essential at successful grafting of *P. falciparum *erythrocytic stages as well as human hepatocytes and *P. falciparum *liver stages in uPA-SCID [50]. Yet, a single clo-lip i.p. injection led to an increase of leukocytes, an increase of the CD43^- ^CD62L^+ ^Ly-6C^+ ^MO subset and an increase of IL-6 and MCP-1. These results suggest that conclusions from studies using clo-lip in mice should be interpreted with care. A third, and likely more satisfactory, strategy relies on the generation of mice with improved genetic deficiency of innate immunity. In this respect, the NOD/SCID/IL2rγ^-/- ^(NOG) mice open new perspectives [1,51], but simultaneously stresses the need for further efforts in the same direction. Indeed, NOG mice are not as good recipient of human skin and artery as SCID/bg mice [15], and, NOG reconstituted with CD34^+ ^haematopoietic stem cells did not sustain the development of human B cells, and most T cells could neither proliferate nor produce IL-2 in response to antigenic stimulation [17]. These results indicate that further improvements in genetic deficiencies are necessary. This in turn implies to better identify the molecular mechanisms critical in graft rejection.

## Conclusions

Taken together data presented in this study show that immunocompromized mice such as NOD/SCID mice in which the number of MP and PMN are controlled to a certain extent by repeated injection of clo-lip and NIMP-R14 antibody respectively, are still able to mount substantial innate defences against xenografts, notably HuRBC and *P. falciparum*. Moreover, the immunomodulatory treatment itself induced inflammatory responses. These results indicate that the use of SCID mice to study human disease need to be carefully interpreted and that further improvements are required to obtain a mouse model fully receptive to grafts of foreign origin.

## Abbreviations

HuRBC: Human red blood cells; MP: macrophage; MO: monocyte; PMN: polymorphonuclear; clo-lip: clodronate-loaded liposomes.

## Competing interests

The authors declare that they have no competing interests.

## Authors' contributions

LA planned and carried out the research, performed experiments and analysed the results, drafted and revised the manuscript. RKT performed experiments and helped to write the manuscript. PM has performed experiments concerning the use of different reagents to improve *P. falciparum *survival. NVR supplied the clo-lip to the lab. JLP was involved in revising the manuscript. PD revised the manuscript and was responsible for overall strategy. All authors read and approved the final manuscript.

## Supplementary Material

Additional file 1 **Comparison of the effects of three anti-PMN monoclonal antibodies**. **(A) **Peripheral blood parasitaemia in 3 different NOD/SCID mice treated either with NIMP-R14 (plain circle, dotted line), RB6-8C5 (open circle) or 1A8 (black square) monoclonal antibody at 10 mg/kg. Black arrows represent injection of HuRBC + clo-clip and one of the three anti-PMN antibodies. **(B) **Peritoneal blood parasitaemia obtained in the NOD/SCID mice treated with different anti-PMN. **(C) **Percentages of CD11b^+ ^Ly-6G^+ ^PMN in mouse peripheral blood following repeated administration of the anti-PMN monoclonal antibodies. Click here for file
