# Supplementary figures and images for: Analysis of innate defences against Plasmodium falciparum in immunodeficient mice
Source: Malar J. 2010 Jul 9;9:197. doi: 10.1186/1475-2875-9-197 (PMC2914061; doi:10.1186/1475-2875-9-197)

**A**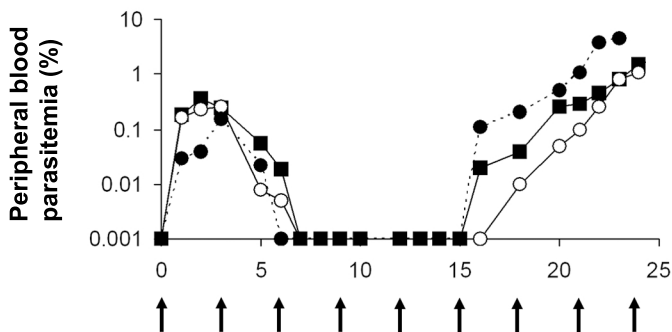**B**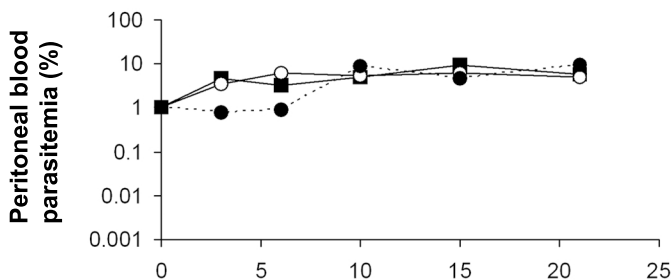**C**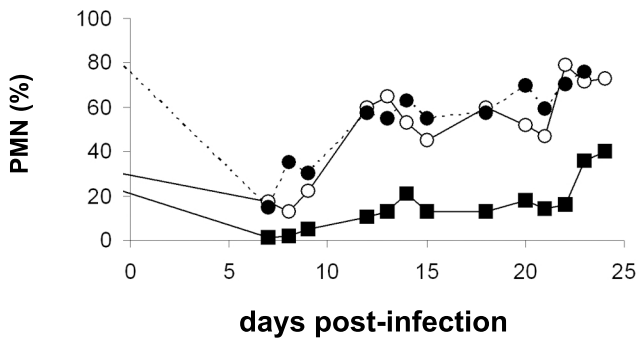

Supplement: Additional file 1 — Comparison of the effects of three anti-PMN monoclonal antibodies. (A) Peripheral blood parasitaemia in 3 different NOD/SCID mice treated either with NIMP-R14 (plain circle, dotted line), RB6-8C5 (open circle) or 1A8 (black square) monoclonal antibody at 10 mg/kg. Black arrows represent injection of HuRBC + clo-clip and one of the three anti-PMN antibodies. (B) Peritoneal blood parasitaemia obtained in the NOD/SCID mice treated with different anti-PMN. (C) Percentages of CD11b+ Ly-6G+ PMN in mouse peripheral blood following repeated administration of the anti-PMN monoclonal antibodies. [file 1475-2875-9-197-S1.PDF]
